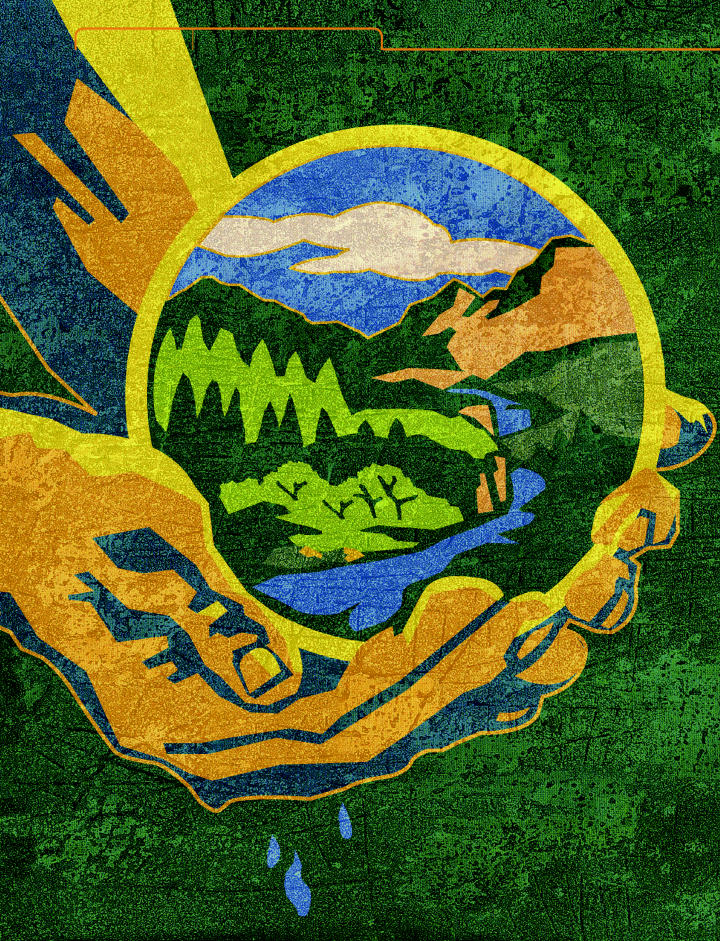# Is Environmental Health a Basic Human Right?

**DOI:** 10.1289/ehp.112-a1006

**Published:** 2004-12

**Authors:** David A. Taylor

Since the Universal Declaration of Human Rights was ratified by United Nations (UN) member countries in 1948, the principle of basic human rights has gained global acceptance. In recent years, proponents of environmental justice have extended that principle into the sphere of the environment, driven by a recognition that increasing scarcity of and conflict over natural resources requires new approaches for securing a peaceful future [see “Global Resources: Abuse, Scarcity, and Insecurity,” *EHP* 112:A168–A175 (2004)]. At the heart of this issue are two key questions: Are the forests, water, air, and food that are essential to our survival common goods to be shared by all? Or are they scarce economic goods, like minerals and timber, that are optimized when they are subject to commercial pressures of supply and demand?

“A human rights argument about natural resources can easily become one extreme of a two-extreme argument,” says Carl Bauer, a research fellow at Resources for the Future (RFF), a nonprofit policy think tank in Washington, D.C. On the one hand, he explains, the term “human right” carries an absolute value that can be hard to trump—it’s like arguing against freedom. At the other extreme is the concept of a free market unhindered by government oversight, which can exert a similar compelling attraction for advocates of a market-driven world economy. Navigating past freighted terms, though, we can examine the factors that shape how we allocate and use natural resources. In a time when the World Bank estimates that more than 1 billion people lack access to safe water, this most essential of resources has become a flash-point in the discussion of human rights versus market forces.

## A Brief History of Rights

In Western society, the concepts of human rights and capitalism both emerged from the European Enlightenment. The English philosopher John Locke (1632–1704) wrote of people’s “natural rights” in terms of a contract between a people and its government. In his 1776 treatise *The Wealth of Nations*, Scottish philosopher and economist Adam Smith described an “invisible hand” that guides markets with a logic of demand and supply.

The term “human right” did not gain broad currency until the last century, and no global consensus existed before the Universal Declaration of Human Rights stated that “all human beings are born free and equal in dignity and rights.” Among its 30 articles, the declaration asserts that everyone has the right to life, liberty, and security of person, and guarantees to all people the right to a standard of living adequate for health and well-being.

That last guarantee has been elaborated in subsequent international agreements, including the 1989 Convention on the Rights of the Child, which states that nations will “recognize the right of the child to the enjoyment of the highest attainable standard of health,” and specifically notes that governments will take measures that account for “the dangers and risks of environmental pollution.” In 2000, the UN Committee on Economic, Social, and Cultural Rights adopted a clarification that extended that right to health to encompass those factors that determine good health, including access to safe drinking water and sanitation. According to the 2003 WHO publication *The Right to Water*, the declaration of water as a human right helps to ensure that governments redress cases of inequitable access to crucial resources. It also means that UN mechanisms for monitoring progress will be used to hold governments accountable.

### Whose Jurisdiction?

To be enforceable, rights must be embedded in fundamental legal documents. In the United States, rights to resources are determined by state and federal law. Carolyn Raffensperger, a lawyer and founding executive director of the nonprofit Science and Environmental Health Network, has reviewed state constitutions and their different mandates on environmental health. In her review, summarized in the December 2003 issue of *Conservation Biology*, she saw a trend exemplified by a few states toward protecting shared resources for current and future generations. Eventually, she says, this trend may inform U.S. constitutional law.

Hawaii’s constitution illustrates this proactive stance. Article XI of the constitution states, “For the benefit of present and future generations, the State and its political subdivisions shall conserve and protect Hawaii’s natural beauty and all natural resources, including land, water, air, minerals and energy sources, and shall promote the development and utilization of these resources in a manner consistent with their conservation and in furtherance of the self-sufficiency of the State. . . . All public natural resources are held in trust by the State for the benefit of the people.”

The state’s supreme court has cited that stewardship role and applied a principle of preemptive precaution against actions that could reasonably be expected to degrade the state’s natural resources. For example, in 2000, the court ruled against a long-standing diversion of an irrigation ditch by sugar plantations of central Oahu. For Raffensperger, it’s then a small step to add to those constitutional protections that all citizens “are impoverished when resource degradation causes a rise in disease.”

Raffensperger concedes that even if the federal government does eventually pursue a similar approach of using preemptive precaution to protect resources, this will not resolve all resource equity problems. Some involve public versus private conflicts, and within private management, there are different situations. “Managing a resource is one thing,” says Raffensperger. “Owning it is another.”

### Ownership versus Management

In the western United States, water rights have long been a bone of contention, with private parties, municipalities, and states squabbling over a region’s rivers for agricultural, industrial, and municipal uses. Recognizing the growing pressures and sinking aquifer levels, the U.S. Department of the Interior recently began an effort called Water 2025 to head off major conflicts and shortages. Water 2025 aims to avert conflicts in part by clarifying rights and legal claims. In that process, the government, armed only with conflict resolution techniques, facilitates dialogue between opposing parties.

In some cases, government contracts with private companies to manage resources such as forests and drinking water have been disastrous. In 1999, for example, the World Bank successfully convinced the city of Cochabamba, Bolivia, to contract out its water supply service. Within months the price of water skyrocketed. Activists claimed the price hikes came because the monopoly emboldened the contractor; the contractor claimed they were caused by rising maintenance and distribution costs. After a violent public outcry, the government quickly reversed its decision.

Despite that ill-fated example, the World Bank continues to see the challenge of providing water to all people as a huge task that requires the combined ingenuity and efficiency of the private sector and government. Others, too, insist there’s no reason to believe that public and private organizations can’t work together to improve equitable access to critical resources. Privately owned water utilities have existed in the United States since the colonial period, says Peter Cook, executive director of the National Association of Water Companies (NAWC), a Washington, D.C.–based industry association. These utilities have a strong record of accountability, efficiency, and health safety, as they are regulated by both the U.S. Environmental Protection Agency and states for quality, and by state public utility commissions to ensure fair customer rates.

Private companies managing public utilities have become more common since the early 1990s. Municipalities have renewed those contracts at a very high rate, suggesting that such public–private partnerships have convincing benefits, says Cook, who adds that private companies can save municipalities 10–40% in operating costs thanks to increased efficiency and lower personnel expenses. As budgets tighten, governments need to consider all available options for providing public services, Cook says.

The NAWC recognizes that poor communities should not endure hardship to pay for safe water. “We think there need to be programs to deal with this,” says Cook. Water bill assistance programs funded by voluntary charitable donations from other customers as well as subsidies from government agencies (such as the NAWC’s proposed Low-Income Water Assistance Program) could help those who need help most, while allowing the utility to charge full cost-of-service rates to all customers. Cook says full cost of service must be charged or the utility will not be economically viable over the long term, with consequences for service, public health, and management of the water resource.

“Just like food, someone has to pay for both the treatment and distribution of finished water before it can be safely consumed,” says Cook. “In the end, it’s the people who actually *own* the resource. We’re mainly concerned with meeting essential human needs for water and sanitation by providing water treatment and distribution services, not resource ownership.”

### The Chilean Experience

Many economists argue that an efficient allocation of resources comes mainly through accurate valuation of scarce resources. They maintain that more careful pricing of a resource—considering nonmarket factors such as social goals of conservation and fair access—will encourage its sustainable use. Economic incentives can open the door to technological innovation and spur better distribution methods.

The kind of subsidy that Cook describes has been used effectively in Chile. In 1981 Chile enacted a water law that promoted free market forces and incentives in water use and reduced government regulation. Chile distinguishes between water use broadly speaking (including industrial, agricultural, and sanitation uses) and the more restricted case of water for people’s survival and health, which includes basic household water.

This is an important distinction, says Bauer, because resources essential for survival should be handled with more concern for equitable access than nonessential resources, which people can choose to forego. The distinction can clarify the policy priority between essential and nonessential water. For basic household water use, Chile’s example shows that targeted subsidies can work. The strength of the Chilean model for household water service, Bauer says, is that it both preserves the larger system of price signals needed for valuing a scarce resource and addresses low-income users’ needs within that system.

“The same price and tariff structures apply to users with different income levels, they’re just dealt with through subsidies,” Bauer explains. “It not only provides transparency about the subsidies provided to poor people—the value is clear from the pricing system—but it also leaves intact the system of price signals that accurately reflect the scarcity of the resource.”

But there are risks in codifying any rigid approach to natural resource access. Says Bauer, “Calling something a human right is well and good, but if a country is too poor to make good on those guarantees, where does that leave you? If the right to low-cost clean water for everyone is politically or economically impossible to enforce, you may end up making the debate about practical issues more difficult instead of furthering it.”

South Africa’s reformed water laws include explicit recognition of equity needs. “This was an important achievement in principle,” Bauer says. “The question is, how can they deliver? It may be better not to lock yourself into a constitutional requirement that everyone knows can’t be met.” On the other hand, he adds, declaring access to be a human right can be an important counterweight to the notion that public interests should be left to the free market.

### Seeking an International Mandate

Unimpressed by market-based efforts to date, environmental justice advocates have sought a human rights tool to leverage change. In April 2004, Earthjustice, a nonprofit public interest law firm based in Oakland, California, called on the UN High Commissioner for Human Rights to take broader action on environmental health problems. In the report titled *Human Rights and the Environment* that was submitted to the commission’s 2004 session in Geneva, Earthjustice developed a proposal based on international human rights agreements and the growing recognition of a link between civil instability and environmental degradation. “The relationship between environmental problems and human rights violations calls for a holistic treatment of these issues,” the report said.

A similar case is made in *Environment and Human Rights*, a 2003 report by Germany’s nonprofit Wuppertal Institute for Climate, Environment, and Energy. That report asserts that commercial resource use—in its extraction of raw materials, ecosystem changes, and pollution effects—has a disproportionately large impact on the poor, who seek only subsistence. The report maintains that sustainability, the pursuit of human rights, and respect for the biosphere—not economic competition—should define a world consensus on allocation of resources.

Perhaps even more urgent than world consensus, though, are choices over priorities among household, agricultural, and industrial uses. Whether or not governments adopt a human rights approach, the debate points up key variables involved in resource allocation and decisions that demand our attention.

## Figures and Tables

**Figure:**